# Initial pH Conditions Shape the Microbial Community Structure of Sewage Sludge in Batch Fermentations for the Improvement of Volatile Fatty Acid Production

**DOI:** 10.3390/microorganisms10102073

**Published:** 2022-10-20

**Authors:** Ylenia Di Leto, Fanny Claire Capri, Antonio Mineo, Alida Cosenza, Giuseppe Gallo, Rosa Alduina, Giorgio Mannina

**Affiliations:** 1 Department of Biological, Chemical and Pharmaceutical Sciences and Technologies, Palermo University, 90128 Palermo, Italy; 2 Engineering Department, Palermo University, 90128 Palermo, Italy

**Keywords:** volatile fatty acid production, sewage sludge, microbiota structure, pH influence, prokaryotic community

## Abstract

Conversion of wastewater treatment plants into biorefineries is a sustainable alternative for obtaining valuable compounds, thus reducing pollutants and costs and protecting the environment and human health. Under specific operating conditions, microbial fermentative products of sewage sludge are volatile fatty acids (VFA) that can be precursors of polyhydroxyalkanoate thermoplastic polyesters. The role of various operating parameters in VFA production has yet to be elucidated. This study aimed to correlate the levels of VFA yields with prokaryotic microbiota structures of sewage sludge in two sets of batch fermentations with an initial pH of 8 and 10. The sewage sludge used to inoculate the batch fermentations was collected from a Sicilian WWTP located in Marineo (Italy) as a case study. Gas chromatography analysis revealed that initial pH 10 stimulated chemical oxygen demands (sCOD) and VFA yields (2020 mg COD/L) in comparison with initial pH 8. Characterization of the sewage sludge prokaryotic community structures—analyzed by next-generation sequencing of 16S rRNA gene amplicons—demonstrated that the improved yield of VFA paralleled the increased abundance of fermenting bacteria belonging to Proteobacteria, Bacteroidetes, Chloroflexi, and Firmicutes phyla and, conversely, the reduced abundance of VFA-degrading strains, such as archaeal methanogens.

## 1. Introduction

Wastewater is the combination of domestic, industrial, urban, and agricultural water collected by urban drainage systems that flows into wastewater treatment plants (WWTPs). In a WWTP, the water undergoes various purification processes, both physical and microbiological, to get rid of organic and inorganic pollutants that can endanger human health and the environment by causing genotoxicity, cytotoxicity, tumorigenesis, and ecotoxicity [1]. Physical processes such as sandblasting (screening), deoiling (grease removal), and grilling (grit removal) represent the first stage of wastewater treatment to eliminate coarse particles. Afterwards, microorganisms with their metabolic capacities degrade the organic matter, producing the simplest compounds and biomass as a result of microbial growth. Clarification and decantation to remove the insoluble component from the water (i.e., sewage sludge including microbial cells) and disinfection represent the two final stages [2]. At the end of the treatment, the purified water is introduced into receiving water bodies, for example, lakes, rivers, and the sea. On the other hand, the produced sewage sludge is, in most cases, disposed of in solid-waste landfills or incinerators. Although conventional WWTPs play an essential role in regulating the eutrophication of aquatic environments, they also cause environmental pollution because of the produced greenhouse gases [3] or the accumulation of sewage sludge disposals.

In Italy, 395,000 tons of dry sewage sludge are produced annually from WWTPs. Various processes are applied to dispose of the sewage sludge (incineration, storage in specific landfills); only 9.9% is reused in agriculture [4]. Thus, it is a challenge to obtain materials and energy still present in the sewage sludge, transforming WWTPs into water resource recovery facilities (WRRFs) or waste water biorefineries (WWBRs) to protect water quality, the environment, and thus human health and for the recovery of high-value compounds with the intent of sustaining circular economic processes [5]. In particular, sewage sludge can be a source of compounds, such as biopolymers or their precursors. The demand for biodegradable polymers has grown by 30%, while that for conventional polymers by 3% [6]. Polyhydroxyalkanoates (PHAs) are thermoplastic biodegradable polyesters with excellent properties. PHAs are environmentally and economically sustainable microbial fermentation products, suitable for replacing petroleum-derived plastics [7].

In a WWTP, microbial communities residing in the sewage sludge use organic matter, such as proteins, polysaccharides, and lipids, and produce VFA, H_2_, and CO_2_ [8,9]. VFA such as acetic, butyric, propionic, and valeric acids can be excellent precursors to feed the production of PHAs that accumulate in bacterial cells in the form of granules as carbon and energy reserves [10]. It is known that the concentration of carbon, temperature, and variation in environmental pH are fundamental parameters that activate the production of PHAs [11]. Due to the importance of VFA production in the development of a PHA-producing facility, an increasing number of studies have been carried out focusing on the prokaryotic (i.e., bacterial and archaeal) microbiota of sewage sludge that affect the production of VFA. Although bacterial and archaeal species have an essential role in VFA yields and many environmental factors influence microbial fermentation [12], studies are now underway to better understand the effects of the microbiota structure on fermentation processes aimed at the production of VFA. Therefore, in order to optimize fermentation processes and the production of VFA, the relationships between different operating conditions leading to the improvement in VFA yields and their effects on microbial communities’ structure need to be deeply analyzed. As an example, Chen et al. (2021) [13] showed that different compositions of hydrothermal conversion wastewater led to the enrichment of different microbial communities, affecting the VFA yields. Other studies have aimed at optimizing the production of VFA of one or more volatile fatty acids by exploiting axenic microbial cultures [14] or by adding specific substances to enhance production [15]. Some studies have been conducted to test the effect of pH on the production of VFA from waste substances (wastewater, kitchen waste, and waste derived from industry and fishing). Many of these studies have found success in producing VFA at acidic pH [16]. Another work showed that comparing acidic and basic conditions the best yields of VFA occur at alkaline pH [17]. To date, the main phyla identified at starting basic pH of waste fermentation are Bacteroidetes, Firmicutes, and Spirochaetes [18]. In a recent work [19], the positive influence of basic pH (i.e., pH 8) was revealed for stimulating the production of VFA. In another very recent study, preliminarily the importance of setting basic pH values at the initial condition to improve the production of VFA was assessed in batch fermentations, then the influence of pH on microbial community structures in continuous fermentation was evaluated comparing pH-controlled and uncontrolled conditions, where pH was set at 7.5–8 [20].

In this study, the sewage collected from a real Sicilian WWTP located in Marineo (Italy) as a case study [21] was used to inoculate batch fermentations to set an optimized production process of VFA. In particular, the production of VFA was evaluated in respect of two initial pH (i.e., pH 8 and 10, respectively). Then, the different levels of VFA due to starting pH conditions were correlated with the structures of the prokaryotic microbiota residing in the sewage sludge in order to highlight the presence of specific prokaryotic members and bioinformatically inferred microbial metabolic capabilities with improved production of VFA.

## 2. Materials and Methods

### 2.1. Experimental Setup

The WWTP is located in Marineo, a town of 6438 inhabitants (ISTAT, 2019) in the metropolitan city of Palermo (Sicily). The plant is located at an altitude of 550 m above sea level and has an average daily flow rate of 2160 m³/d. The WWTP has a sewage sludge scheme, whereby the incoming wastewater is first subjected to sandblasting, deoiling, and mud screening, then subjected to the sewage sludge process [21]. In recent studies, it has been found that the basic pH 10 set at the start of batch fermentation favors the production of VFA [19]; thus, two experiments in sequence were carried out (reported as conditions A and B, respectively) to evaluate the effect of pH 8 and pH 10, respectively. The sewage sludge inoculum samples (ten liters each) for experiments A and B were collected 24 days apart from each other and were labeled T0-A and T0-B, respectively (Table 1). The two sewage sludge samples—collected from the recirculation circuit that carries the biomass from the settler to the biological sewage sludge reactor inside the plant—were transported to the laboratory at room temperature. The batch fermentation test reactors were assembled in anaerobiosis, on the same day of the sampling. For each tested pH value, 8 and 10, which are referred to as experiments A and B, respectively, two 1.1 L batch reactors were assembled in parallel in the following way: one having the pH adjusted by adding a saturated solution of NaOH 1N at the beginning of fermentation at the pH 8 (Experiment A) or at pH 10 (Experiment B), and the other one with unadjusted pH used as the control condition (Control A or Control B, respectively). Thus, a total of four batch reactor fermentations were performed (Table 1). The reactors are magnetic stirred glass bottles, equipped with two sampling ports for liquid and gas sampling and two electrode ports. pH, oxidation-reduction potential (ORP) and temperature were continuously monitored using the relative probe connected to a Multi 3630 IDS WTW multimeter (Xylem brand). The fermentation processes were carried out until the soluble chemical oxygen demand (sCOD) and pH values tended to be almost stabilized. Fermentation took place at room temperature for 15 days. The reactors were constantly monitored.

### 2.2. sCOD and VFA Analysis

Once the batch fermentation tests were assembled and equipped with a probe capable of detecting the pH, the soluble chemical oxygen demand (sCOD) was measured each day. The samples for which sCOD and pH were monitored were Control A, Control B, Experiment A and Experiment B. For these analyses, the sewage sludge sample was filtered each time and the analyses of sCOD were carried out on the supernatant. Sampling involved the use of a syringe through which 20 mL of each sample was collected from each batch fermentation reactor every day and placed in sterile 50 mL tubes. The samples were then subjected to centrifuge at 4000× *g* for 5 min, then the supernatant was filtered with sterile 0.45 μm Millipore filters. The filtrate was collected in new sterile tubes and subjected to analysis. Analyses of sCOD on the filtrate were carried out using a COD Cell Test—photometric 25–1500 mg/L kit (Supelco) following the manufacturer’s instructions. For the photometric reading, quartz cuvettes and a Merck spectrophotometer were used. On the peak day of the sCOD, the filtrate taken from the batch fermentation test was subjected to VFA analysis to evaluate the production. The VFA analyses were carried out in a gas chromatograph (Agilent Technologies 7820A, Santa Clara, CA, USA) equipped with a flame ionization detector (FID) and a DB FFAA column (30 m × 0.25 × mm × 0.25 µm). The filtrate samples with 0.45 micrometer syringe filters (Sartorius Minisart) were prepared by adding dimethyl carbonate (DMC Reagent Plus, Sigma Aldrich, St. Louis, MI, USA). A rate of about 1 uL through a 10 uL Hamilton microsyringe was analyzed. Samples (200 μL) of the filtered fermentation mixture samples were transferred into gas chromatography (GC) vials and 800 μL of a stabilizing solution composed of HgCl_2_ (0.5 g), phosphoric acid (5 mL, 100%) and hexanoic acid (0.54 mL, internal standard) was subsequently added. A gas chromatograph (GC) (Agilent Technologies 7820A), equipped with a flame ionization detector (FID) and a DB FFAA column (30 m × 0.25 × mm × 0.25 µm), was used for the detection of short-chain fatty acids [19].

### 2.3. Prokaryotic Microbiota Structure Analysis

Analysis of prokaryotic microbiota structure of sewage sludge was performed by metataxonomics based on NGS analysis of 16S rRNA gene amplicons obtained from metagenomic DNA. In particular, a total of six sewage sludge samples were analyzed, as indicated in Table 1: T0-A and T0-B corresponding to the sewage sludge collected in Marineo WWTP [21]; Control A and Control B corresponding to the sewage sludge of batch reactors in which pH was not adjusted at the beginning of the process; Experiment A and Experiment B corresponding to the sewage sludge of the batch reactors in which the pH was set at the beginning of the fermentation at pH 8 and pH 10, respectively. The Control A, Control B, Experiment A, and Experiment B samples were collected at the end of the fermentation process.

The analyses were performed on unfiltered and uncentrifuged samples. The procedures already described [19] were followed. The DNA extractions were evaluated by 1% (*w*/*v*) agarose gel electrophoresis analysis, with the addition of 0.5 μg/mL ethidium bromide for visualization using a UV lamp. The concentrations of the DNA, extracted from 1 g of sewage sludge samples and the corresponding tenfold serial dilutions were measured by reading absorbance at 260 nm with a NanoDrop 2000c spectrophotometer (Thermo Fisher Scientific, Waltham, MA, USA). The purity of the extracted DNA was evaluated by measuring the absorbance ratios (260/280 and 260/230 nm) to indicate contamination due to proteins and organic compounds or chaotropic agents, respectively. The following primers were used for PCR amplification of the V3-V4 regions of the 16S gene: PRO 341F CCTACGGGNBGCASCAG and PRO 805R GACTACNVGGGTATCTAATCC. For PCR, DreamTaq DNA polymerases (ThermoFisherScientific) was used following the manufacturer’s instructions. The thermal profile used was: 95 °C for 2 min; 35 cycles of denaturation at 95 °C for 30 s, annealing at 55 ° C for 30 s, elongation at 72 °C for 30 s; final elongation at 72 °C for 5 min. Amplification products were sequenced in one 300 bp paired-end run on an Illumina MiSeq platform at BMR Genomics (Padova, Italy). The raw 16S rDNA data were processed by using the QIIME2 software (https://qiime2.org/ (accessed on 30 January 2022)) as paired-end sequences. In the denoising approach, overlapping paired-end reads were processed with the plugin DADA2. Unique amplicon sequence variants (ASVs) were assigned and aligned to the Greengenes reference database at 99% sequence similarity (https://greengenes.secondgenome.com/ (accessed on 30 January 2022)). For each sample, the number of ASVs and the percentages of relative abundances of phyla, orders, classes, and families were determined. Principal coordinate analysis (PCoA) was chosen as a multivariate statistical approach and was generated starting from the Bray–Curtis distance matrix using Emperor software. A heatmap was obtained through the online web server (http://heatmapper.ca/expression/ (accessed on 8 January 2022)) based on genera. The heatmap was generated by a “complete linkage” calculation using Spearman rank correlation.

METAGENassist (http://www.metagenassist.ca (accessed on 6 January 2022)) was used to distinguish the microbial species based on their metabolic activity [22].

## 3. Results and Discussion

### 3.1. sCOD and pH Values in Batch Fermentations

**Figure 1:**
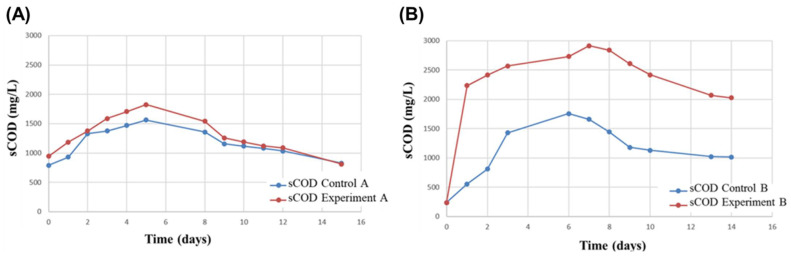
sCOD (mg/L) variation for 14–15 days in Condition A (**A**) and Condition B (**B**) batch fermentations. The red lines represent the controls (Control A and Control B), while the green lines represent the experiments (Experiment A and Experiment B).

The sewage sludge collected in the WWTP of Marineo had the following parameters: pH 7.2 and sCOD 790 mg/L (T0-A), and pH 7.2 and sCOD 237 mg/L (T0-B). Table 2 reports the data constantly monitored for “Control A” and “Experiment A” samples from the batch fermentations of Condition A. Table 3 reports the data for “Control B” and “Experiment B” samples from the batch fermentations of Condition B. Figure 1 shows graphs relating to the sCOD along with the incubation on batch reactors and highlights the peak per day.

As shown in Figure 1, the sCOD peak was reached on the fifth day of incubation of Experiment A and Control A, on the sixth day for control B, and on the seventh day for experiment B. In addition, the amount of sCOD obtained in Experiment B was the highest measured, reaching 3000 mg/mL. The other samples reached approximately 1500–1800 mg/mL. The production of sCOD increased by 1.5-fold at pH 10 over pH 8 (2020 mg/L against 1391.71 mg/L). This result confirms the positive effect of basic pH at the beginning of fermentation on microbial species leading to high amounts of sCOD. VFA amount, determined by GC analysis, confirmed high levels on the peak day of the sCOD. Table 4 reports both the values of VFA and the respective VFA/sCOD ratios.

As revealed by previous studies, sCOD and the production of VFA are affected by pH [19,23,24]. In particular, enhanced fermentation, and thus VFA production at pH 10 in a shorter time than at pH 8 was observed [23]. Comparing these results with other recent studies in which acid-forming batch fermentation was also performed at pH 10 for 15 days, such as in Atasoy et al. (2020) [24] (i.e., 1942 mg COD/L), it is possible to note that the maximum VFA yield reported here (2020 mg COD/L) is higher. It is also interesting to note that despite the initial differences, the pH values are similar in all reactors during batch fermentations, starting from the second day onwards, similarly to the observed results of Atasoy et al. (2020) [24] for mixed culture fermentations. This could be due to the metabolic versatility of microbial communities in mixed cultivations, in which acidification processes could be counteracted by the metabolic activity of other microbial members acting as a metabolic consortium, as was also suggested by Wang et al. (2013) [25].

### 3.2. Sequencing Output and Analysis

Metataxonomic analyses—based on NGS analysis of 16S rRNA gene amplicons obtained from metagenomic DNA of sewage sludge—were conducted using the samples collected from both sets of the two batch fermentation reactors. In total, 202,731 high-quality reads were filtered from the 545,624 raw reads obtained. A total of 1683 ASVs were identified using the suite QIIME2, after the processes of filtration, denoising, and merging (Table 5). ASVs were classified at the genus level using a 99% sequence similarity threshold against the Greengenes database.

Rarefaction curves (Figure S1, Supplementary Material) showed a good level of diversity sampling, as confirmed by Good’s coverage index for all the samples, with an average of 0.99. The rarefaction curves based on the comparison of ASV abundance and the number of sequences analyzed showed that samples tended to reach a saturation plateau, thus demonstrating that the analyses were representative of the communities under investigation.

### 3.3. Structure of Prokaryotic Communities of Sewage Sludge

Most ASVs have been assigned to the Bacteria and only about 2% to Archaea domains. Therefore, the prokaryotic composition of sewage sludge from conditions A and B was comparatively analyzed considering the relative abundances at the phyla level firstly and then at the level of family and finally at the level of genus.

**Figure 2:**
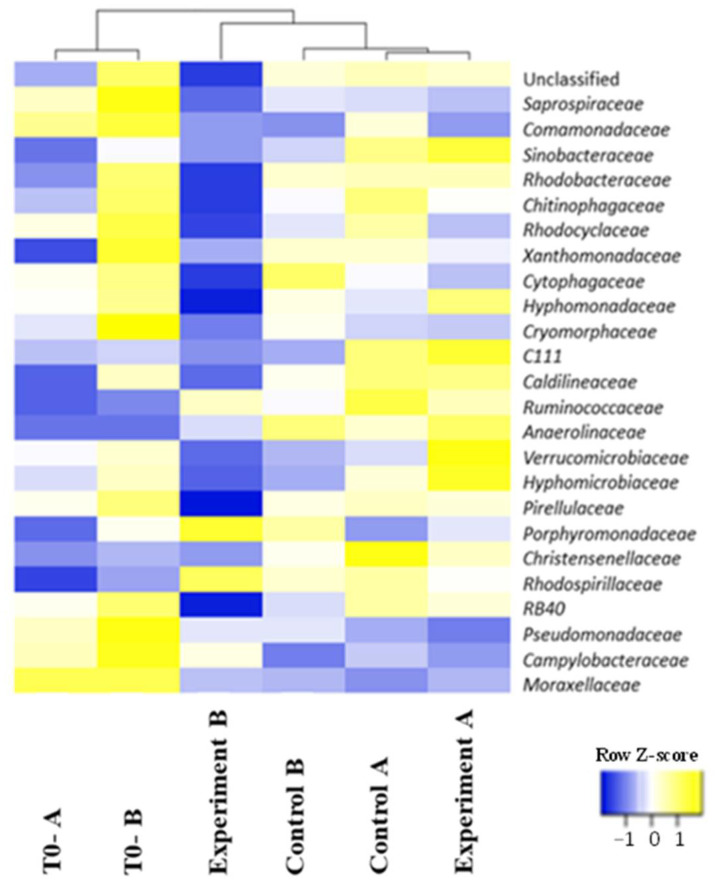
Heatmap of 25 most abundant prokaryotic families’ composition profiles. The color intensity in each sample is normalized to represent its relative ratio in the two groups. Colors from blue to yellow indicate the relative values of microbiota.

First of all, heatmap analysis (Figure 2) clearly showed that the T0-A and T0-B samples were more similar to each other than the others. In addition, while the Control A and Experiment A samples had a very similar microbial community and tended to cluster with Control B samples, Experiment B was separated from the other samples. This result clearly indicates a role of starting pH value in modulating microbial components, thus possibly positively influencing the production of VFA.

**Figure 3:**
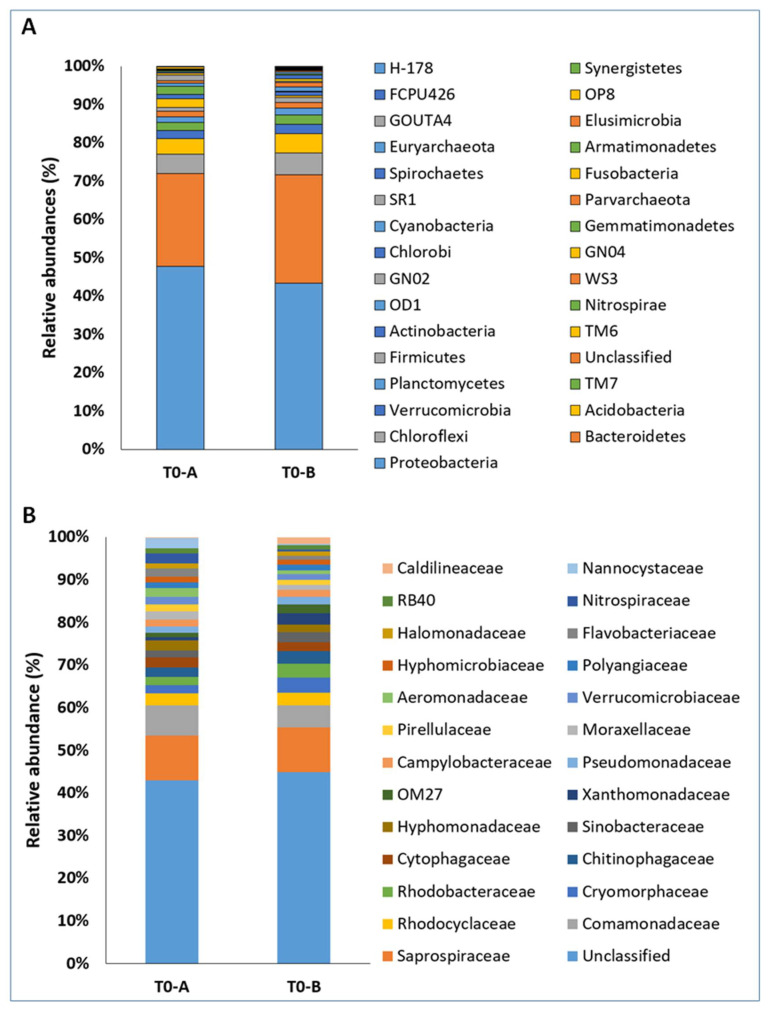
Prokaryotic community structures of T0-A and T0-B sewage sludge samples at the level of phylum (**A**) and family (**B**). In panel B, the top 25 most abundant families are reported. Alphanumeric codes refer to candidate divisions.

The microbial composition of T0-A and T0-B showed no substantial difference at phyla or family level (Figure 3). The most represented phyla were Proteobacteria, Bacteroidetes, Chloroflexi, and Acidobacteriota, and Saprospiraceae, Comamonadaceae, and Rhodocyclaceae were the most represented families in all samples. The percentage abundances of the various phyla—more specifically, of the families that made up the T0-A and T0-B samples—were similar.

**Figure 4:**
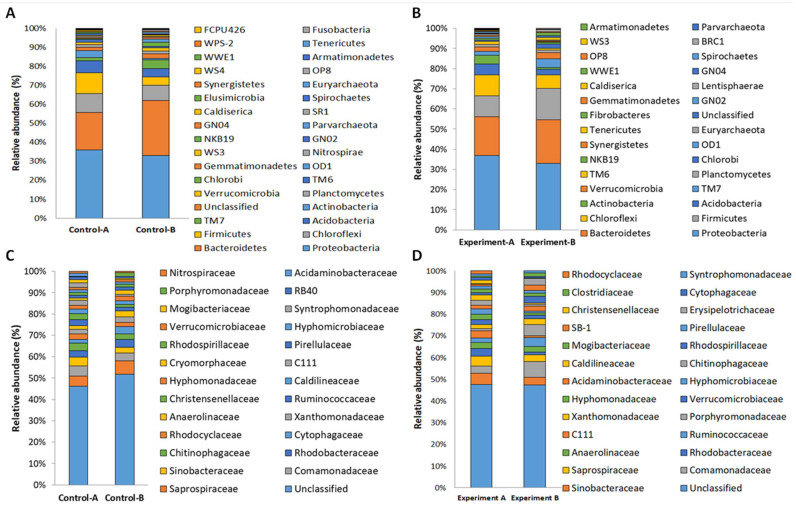
Prokaryotic community structures at the level of phyla (**A**,**B**) and family (**C**,**D**) of sewage sludge samples collected from Control A, Control B, Experiment A, and Experiment B fermentations. In (**C**,**D**), the top 25 most abundant families are reported. Alphanumeric codes refer to candidate divisions.

In all samples of the four batch fermentations, the most dominant phyla were Proteobacteria and Bacteroidetes, followed by Chloroflexi and Firmicutes (Figure 4A,B). The presence of Proteobacteria and Bacteroidetes as the main phyla is in agreement with the report of Guo et al. (2017) [26], even if a very low representation of Firmicutes and no Chloroflexi were revealed in the latter. Interestingly, the majority of bacteria belonging to these phyla have been reported to be capable of degrading complex organic matters for the production of VFA [27,28]. During anaerobic fermentations, a crucial role was assigned to sewage sludge Proteobacteria and Bacteroidetes for propionate, butyrate, and acetate metabolism [29]. Interestingly, it has been observed that some species of Firmicutes, such as *Bacillus megaterium*, can assimilate glucose, acetic acid, butyric acid, and caproic acid and produce PHA with yields of 9–11% of the dry cell weight [30]. Thus, the higher percentage of Firmicutes in the batch fermentation test at pH 10 could justify the highest VFA values. This is even more evident from the most abundant families that were detected by the analysis (Figure 4C,D). The most abundant family was Saprospiraceae, followed by Comamonadaceae, Sinobacteraceae, and Rhodobacteraceae. There were no relevant family differences between the two batch fermentation tests. Saprospiraceae were more abundant in both T0 samples and gradually decreased in control and experiment samples in both batch fermentations and assumed an abundance profile similar to the Bacteroidetes phylum to which they belong. Members of the Saprospiraceae family were detected in sewage sludge samples, but their possible role in wastewater treatment is not well elucidated. Probably, Saprospiraceae members may be important in breaking down complex carbon sources. It has been suggested that their hydrolytic activity provides simple carbon substrates for other microorganisms involved in nutrient removal, and this could also be the limiting step for these processes [31]. For Comamonadaceae, there was a slight increase in relative percentage in the Experiment B sample compared to the corresponding control condition B (5.8% and 2.8%, respectively). This bacterial family not only has fermenting and PHA production capabilities but also its members are known denitrifying agents of sewage sludge microbial communities, as they are able to simultaneously remove nitrates and toxic organic contaminants from the wastewater [32].

**Figure 5:**
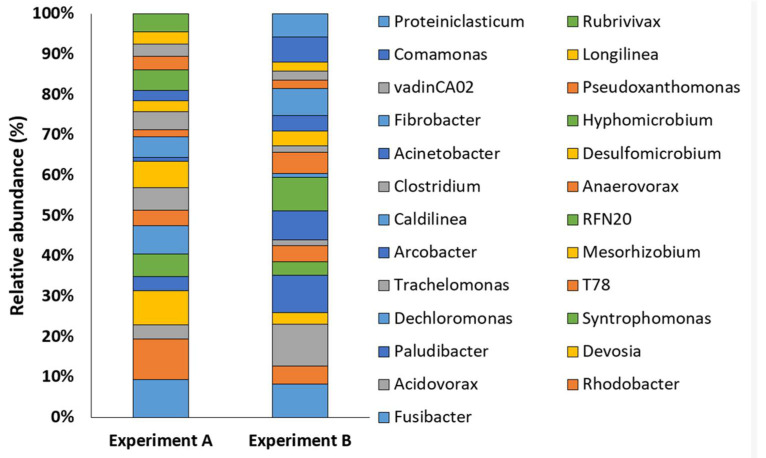
Prokaryotic community structures of sewage sludge samples collected from Experiment A and Experiment B fermentations reporting the top 25 taxa at the genus level. Alphanumeric codes refer to candidate divisions.

In order to obtain a deep understanding of the relationships between sewage sludge microbial community structure and the production of VFA, bacterial and archaeal genera residing in sewage sludge microbiota were compared. Among the 25 most abundant genera, only bacterial members were highlighted, being archaeal genera scarcely represented (Figure 5).

The main bacterial genera found in the highest percentage in all the analyzed samples were *Rhodobacter, Trachelomonas, Dechloromonas, Devosia,* and *Pseudomonas.* However, by examining the samples individually, it was possible to notice a different distribution of genera. For example, in Experiment B, characterized by the highest yield of VFA, the main bacterial genera present were *Acidovorax* (2.47%), *Paludibacter* (2.18%), *Fusibacter* (1.93%), *Arcobacter* (1.69%), *Comamonas* (1.45%), *Pseudomonas* (1.15%) and *Acinetobacter* (0.90%). These bacterial genera were also present in the other samples, but reached the highest percentage in Experiment B. Interestingly, previous studies demonstrated not only the ability of *Acidovorax* to survive in basic pH conditions till pH 12 but also that the basic pH creates a selective pressure in favor of this bacterial genus. At basic pH and anaerobic conditions, *Acidovorax* is an excellent producer of VFA [33]. Strains belonging to the *Comamonas* (1.45%) genus are not known for fermentation capacity. However, in a previous study by Zakaria et al. (2009) [34], it was found that *Comamonas* strains have excellent growth in the presence of VFA released by other bacterial strains, especially in the presence of acetic, propionic, and butyric acids. *Acidovorax* and *Comamonas* species are mainly involved in the storage process of PHAs, starting from volatile fatty acids [35]. Another strongly represented genus in the Experiment B sample was *Paludibacter* (2.18%). Strains belonging to this bacterial genus have the ability to use various sugars for the production of VFA, especially propionate and acetate. The strain has an optimum pH of 6.6–7 but also resists much higher pH, for example, pH 10 [36].

Other experiments that test the production of VFA at different pH conditions confirm that the condition in which the system is most performing is pH 10. At this pH, the genus *Pseudomonas* is likely to be the main producer of acetic and propionic acid starting from organic carbon in the sludge [37]. In the Experiment B sample, the genus *Pseudomonas* is present in a modest percentage (1.15%) while it seems that in the T0-B the genus is more represented. *Fusibacter* present in Experiment B sample with a high percentage (1.93%) is an excellent producer of VFA in anaerobiosis [38] as *Arcobacter* (1.69%), which simultaneously manages to degrade the biosolids of the sludge and fermenting the organic matter into VFA [39]. Finally, *Acinetobacter* (0.90%) has fermentation capacity, especially in the production of acetic acid [40]. Thus, the initial pH 10 exerted selective pressure in favor of the genera *Acidovorax*, *Paludibacter, Fusibcter, Arcobacter, Comamonas, Pseudomonas, and Acinetobater* in the Experiment B sample and are excellent producers of VFA. This suggests that pH 10 created the most performing conditions to produce VFA. This is also confirmed by the fact that in the Experiment A sample (pH 8), which gave lower yields of VFA (Figure 1); these genera were present in a reduced percentage or are not present at all. However, the genera *Rhodobacter* (1.51%), *Fusibacter* (1.41%), *Devosia* (1.27%), *Dechloromonas* (1.06%), and *Mesorhizobium* (0.98%) were present in the Experiment A sample. *Fusibacter* was present at both pH 10 (1.93%) and pH 8 (1.41%). However, another work demonstrated how controlled pH initially promotes the degradation of VFA by *Rhodobacter* for the production of H_2_ [41]. The genus *Devosia* (1.27%) is also involved in the H_2_ production process, degrading the VFA for acetogenesis [42]. Hence, *Devosia,* like *Rhodobacter,* is involved in the consumption of VFA produced by the other strains. *Dechloromonas* (1.06%) and *Mesorhizobium* (0.98%) are not only known to be capable of producing VFA but also of producing and accumulating PHA [43,44]. Therefore, the lower yield of VFA relative to the Experiment A sample (pH 8) may be due to the selection of specific microbial genera that are able to produce VFA, while others degrade them.

The control of operating parameters is an important aspect of a microbial fermentation process to achieve the product or perform the reaction of interest and the knowledge of the way in which operating parameters control and shape microbial community structure can be exploited for improving the process. In particular, the association between fermentation conditions leading to high levels of VFA and the presence of specific members of microbial community structure of sewage sludge can be exploited to drive reshaping of community structures in such a way to maximize and to inhibit the presence of microbial producers and consumers, respectively. This could be achieved in different ways that, for example, involve the manipulation of sewage sludge used as inoculum. As an example, sewage sludge can be subjected to pretreatment procedures that affect the relative abundance or interfere with the metabolic activity of specific members, as described in the study of Muhorakeye et al. (2022) [20] where sodium 2-bromoethane sulfonate was used to reduce the number of methanogen members that are known to be VFA consumers. Another possibility is to perform a pre-inoculum step in which bacterial growth is performed controlling operational parameters, such as temperature, oxygen [45], and pH [20] to obtain a restructured sewage sludge enriched with specific members to be then used as inoculum in fermentation reactors for production of VFA.

In addition, in silico analysis of metabolic functions can be useful to highlight which metabolic processes can be stimulated or reduced. The following paragraph highlights the metabolic activities that can be positively or negatively associated with production of VFA based on the relative abundance of microbial taxa residing in the sewage sludge of batch fermentations.

### 3.4. Metabolism and Oxygen Requirement Analysis

**Figure 6:**
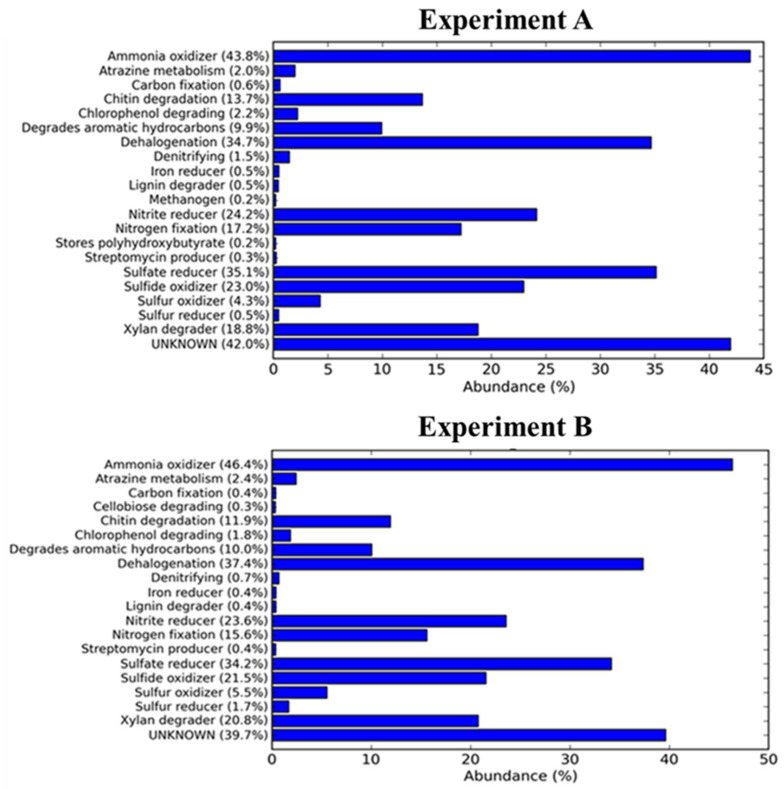
Putative metabolic requirements of the sewage sludge prokaryotic communities of Experiment A and Experiment B batch fermentation.

Based on the interference of taxonomic-to-phenotypic mapping of metabolism, bacteria and archaea were classified on the basis of their oxygen demand and metabolism by [22]. The aim of the metabolic study was to identify whether there are microorganisms capable of producing PHA from VFA and analyze the diffusion of methanogenic strains, to correlate their activity with pH and the presence of VFA. In this study, all samples contained aerobic and anaerobic microbial strains. This is possible because first of all the sewage sludge samples come from an aerobiotic environment, and secondly because even if the fermentation takes place in anaerobiosis, when the batch fermentation reactor is assembled there is a quantity of oxygen that can be consumed by microbial cells as the fermentation proceeds, up to complete anaerobiosis. It was found that in the Experiment A samples 23% of prokaryotic species were aerobic and 16.2% were anaerobic. In the experiment B batch fermentation samples, there was a slight increase of both aerobic and anaerobic strains (25% and 19.6% respectively). On the basis of the type of metabolic features, all samples contained ammonia oxidizers, dehalogenating members, nitrite reducers, and sulfate reducers (Figure 6). The prokaryotic communities of the two conditions A and B have similar metabolic profiles, but in Experiment A, a higher representation of methanogenic microbial members and strains performing denitrification and chitin degradation, and, on the contrary, a lower representation of sulfur-reducing strains and strains degrading cellobiose and storing polyhydroxybutyrate were found. On the basis of this result, the lower concentration of VFA in Experiment A may be due to the presence of methanogenic microbial strains. In fact, methanogens, that are prokaryotic microorganisms belonging to the Archaea domain, have an optimal pH range around neutrality and may consume VFA to produce CH_4_ [46]. In addition, the reduced amount of cellobiose degraders may be related to a reduced availability of carbohydrates since cellobiose is the product of microbial hydrolysis of cellulose, one of the most abundant sources of carbon in different ecosystems, including wastewater [47,48].

Thus, the analysis of prokaryotic community structures shaped by pH conditions revealed the positive and negative selection of VFA producers and consumers, respectively, as well as the in silico prediction of metabolic functions—based on prokaryotic community structures—suggest that the development of fermentation conditions inhibiting methanogenesis and/or stimulating carbohydrate utilization can be a further practicable strategy to improve the production of VFA [49,50].

## 4. Conclusions

The investigation of the association between fermentation conditions and microbial community structure should be considered as the basis to promote the establishment of sustainable and feasible processes to get high-value products, such as VFA. In this work, two batch fermentation conditions, namely, Condition A and Condition B, were analyzed (Table 1): the former in which the starting pH was initially set at 8 and the latter in which the initial pH was set at 10 in comparison with unadjusted pH. In a previous study by Presti et al. (2021) [19], it was shown that basic pH positively influences the production of VFA. In this study, it was further demonstrated comparatively that the higher basic is the starting pH, the higher production of VFA is observed. In addition, this finding parallels the restructure of the sewage sludge prokaryotic community residing and operating in batch fermentation reactors. Despite the pH difference being set at the beginning of batch fermentation, the production of VFA on the peak day of the sCOD was significantly higher in the Experiment B sample than in the others. The metataxonomic based on NGS analysis of the 16S rRNA gene showed that the sewage sludge samples collected from Marineo WWTP [21] have similar prokaryotic community compositions, although they were collected within 24 h of each other. In addition, in the sewage sludge of Experiment B, in which the initial pH was set at 10, the microbial community composition evolved in a different way during batch fermentation in comparison with all of Experiment A, in which the initial pH was set at 8, and the unadjusted-pH control tests. Thus, it can be surmised that pH 10 shaped the microbial community structure favoring the fermenting microorganisms and thus increasing the production of VFA. The production of VFA by fermentation processes is positively correlated with the abundance of Firmicutes, Bacteroidetes and Chloroflexi in the microbial community, as observed in Experiment B. Accordingly, these three phyla have already been shown involved in the production of VFA from food waste at pH 10 by Khatami et al. (2021) [51]. Thus, it is evident that the initial pH 10 of the batch fermentation test promotes the growth of all those bacteria and archaea with fermentation capacity and a maximized production of VFA. Indeed, as it has been highlighted by the analysis of putative metabolic features of the sewage sludge prokaryotic communities of Experiment A and Experiment B, the starting pH 10 promotes a reduction of microbial members that use VFA to produce other substances (such as, methanogens that produce CH_4_). Therefore, this work sheds light on the fact that the evolution and selection of the microbial community residing in sewage sludge of WWTP can be driven in favor of VFA producers by setting the starting parameters of operating conditions. In addition, these results suggest that the development of operating and fermentation conditions affecting microbial metabolism can be a practicable strategy to improve the production of VFA. In perspective, this study suggests the possibility to scale up the process to maximize the production of VFA to sustain PHA production. Thus, the exploitation of a high-yielding microbial community coupled with the use of optimized fermentation conditions could be a strategy to favor the development of PHA-based bioplastics and start from waste for a “greener” world.

## Figures and Tables

**Table 1:** Samples analyzed in this study.

Condition	Sample	Sampling Date	Sample Details
Condition A (pH 8)	T0-A	01/06/2021	Sewage sludge sample collected from Marineo WWTP and used as inoculum for Control A and Experiment A batch fermentations
	Control A	16/06/2021	Sewage sludge sample collected after batch fermentation without pH adjustment
	Experiment A	16/06/2021	Sewage sludge sample collected after batch fermentation with initial pH adjustment at 8
Condition B (pH 10)	T0-B	25/06/2021	Sewage sludge sample collected from Marineo WWTP and used as inoculum for control B and experiment B batch fermentations
	Control B	9/07/2021	Sewage sludge sample collected after fermentation without pH adjustment
	Experiment B	9/07/2021	Sewage sludge sample collected after fermentation with initial pH adjustment at 10

**Table 2:** Average sCOD and pH of the Control A and Experiment A batch fermentations of Condition A experiments.

Batch Fermentation Condition A—Initial pH 8					
Control A			Experiment A		
Day	sCOD mg/L	pH	Day	sCOD mg/L	pH
0	790 ± 16	7.2 ± 0.3	0	790 ± 16	8 ± 0.8
1	930 ± 18	6.8 ± 0.2	1	1186 ± 8	6.8 ± 0.5
2	1329 ± 25	6.6 ± 0.1	2	1377 ± 10	6.7 ± 0.3
3	1374 ± 26	6.6 ± 0.1	3	1587 ± 15	6.6 ± 0.2
4	1468.5 ± 25	6.6 ± 0.1	4	1705.5 ± 15	6.6 ± 0.1
5	1563 ± 34	6.6 ± 0.1	5	1824 ± 22	6.6 ± 0.1
8	1359 ± 14	6.6 ± 0.1	8	1540.5 ± 27	6.8 ± 0.1
9	1155 ± 17	6.6 ± 0.1	9	1257 ± 25	6.8 ± 0.1
10	1117.5 ± 19	6.7 ± 0.1	10	1188 ± 23	6.7 ± 0.1
11	1080 ± 20	6.7 ± 0.1	11	1119 ± 18	6.7 ± 0.1
12	1035 ± 18	6.7 ± 0.1	12	1089 ± 20	6.8 ± 0.1
15	825 ± 15	6.7 ± 0.1	15	813 ± 15	6.8 ± 0.1

**Table 3:** Average sCOD and pH of the Control B and Experiment B batch fermentations of Condition B experiments.

Batch Fermentation Condition B—Starting pH 10					
Control B			Experiment B		
Day	sCOD mg/L	pH	Day	sCOD mg/L	pH
0	237 ± 6	7.2 ± 0.3	0	237 ± 6	10 ± 2
1	556 ± 15	6.8 ± 0.2	1	2234 ± 31	7.4 ± 1
2	809 ± 14	6.6 ± 0.1	2	2413 ± 28	6.7 ± 0.4
3	1428 ± 20	6.6 ± 0.1	3	2568 ± 24	6.6 ± 0.1
6	1756 ± 26	6.6 ± 0.1	6	2733 ± 36	6.7 ± 0.1
7	1661 ± 30	6.6 ± 0.1	7	2916 ± 34	6.8 ± 0.1
8	1444 ± 24	6.6 ± 0.1	8	2838 ± 32	6.8 ± 0.1
9	1180 ± 19	6.6 ± 0.1	9	2610 ± 29	6.8 ± 0.1
10	1131 ± 17	6.7 ± 0.1	10	2418 ± 27	6.8 ± 0.1
13	1023 ± 21	6.7 ± 0.1	13	2070 ± 34	6.8 ± 0.1
14	1014 ± 23	6.7 ± 0.1	14	2025 ± 36	6.8 ± 0.1

**Table 4:** Average VFA determination by GC assay and relative VFA/sCOD ratios of Condition A and Condition B batch fermentations.

Batch Fermentation	Peak Day of sCOD	VFA mg COD/L	VFA/sCOD
Control A	5	1303.54 ± 54.07	0.834
Experiment A	5	1391.71 ± 27.66	0.763
Control B	6	1411.82 ± 28.02	0.8
Experiment B	7	2020 ± 37.04	0.69

**Table 5:** Results of bioinformatic analysis of NGS reads obtained from metataxonomic analysis of sewage sludge samples collected from Condition A and Condition B batch fermentations.

Sample	Input	Filtered	Percentage of Input Passed Filter (%)	Denoised	Merged	Percentage of Input Merged (%)	Nonchimeric	Percentage of Input Nonchimeric (%)
T0-A	74,926	61,752	82.42	56,341	45,171	60.29	28,642	38.23
Control A	107,349	88,438	82.38	82,197	66,368	61.82	40,084	37.34
Experiment A	101,116	81,999	81.09	76,364	63,186	62.49	37,502	37.09
T0-B	117,728	96,462	81.94	89,387	75,002	63.71	46,400	39.41
Control B	83,492	67,136	80.41	61,455	51,157	61.27	32,491	38.92
Experiment B	61,013	51,049	83.67	46,841	40,321	66.09	18,048	29.58

## Data Availability

All data are contained within the article or supplementary material. GS data have been registered with the BioProject database with the accession identifiers PRJNA752593 and PRJNA890427 or are available on request to the corresponding authors.

## Supplementary Materials

The following supporting information can be downloaded at: https://www.mdpi.com/article/10.3390/microorganisms10102073/s1. Figure S1: Rarefaction curves on sequencing data obtained from different compartments.
