# Supplementary material for: Initial pH Conditions Shape the Microbial Community Structure of Sewage Sludge in Batch Fermentations for the Improvement of Volatile Fatty Acid Production
Source: Microorganisms. 2022 Oct 20;10(10):2073. doi: 10.3390/microorganisms10102073 (PMC9611766; doi:10.3390/microorganisms10102073)
Supplement: Supplementary file 1 [file microorganisms-10-02073-s001.zip › microorganisms-1971945-supplementary.pdf]

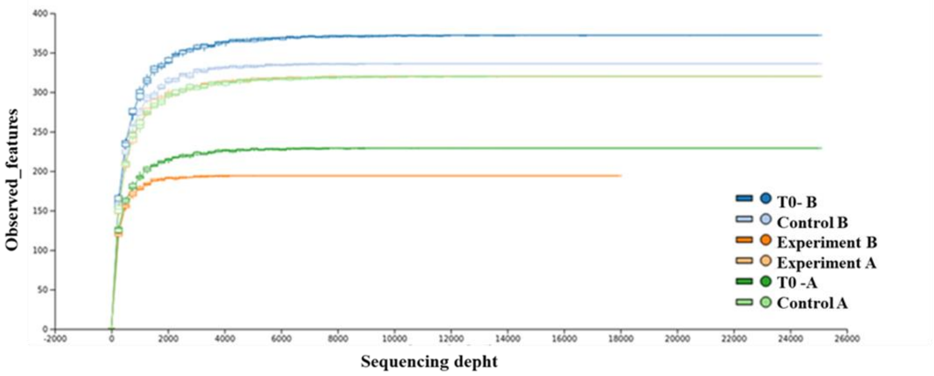

Figure S1. Rarefaction curves on sequencing data obtained from different compartments. The number of observed characteristics (representative of the ASVs) found in each sample is reported as a function of the sequencing effort. The asymptotic trend of the curves indicates that the number of readings generated is representative of the entire community.
